# Preventive and Therapeutic Effects of Hydrogen-Generating Si-Based Agent on Pressure Ulcers in Mice

**DOI:** 10.3390/biomedicines13102475

**Published:** 2025-10-11

**Authors:** Naoya Otani, Takaki Oue, Yuki Kobayashi, Hikaru Kobayashi, Koichi Tomita, Tateki Kubo

**Affiliations:** 1 Department of Plastic Surgery, The University of Osaka Graduate School of Medicine, Osaka 565-0871, Japan; 2 SANKEN, The University of Osaka, Osaka 567-0047, Japan; 3 Department of Plastic and Reconstructive Surgery, Kindai University Faculty of Medicine, Osaka 589-8511, Japan

**Keywords:** pressure ulcer, hydrogen, oxidative stress, inflammation, apoptosis

## Abstract

**Objectives**: As a known antioxidant, hydrogen has been useful for treating pressure ulcers. However, conventional methods of hydrogen administration have limitations with regard to dosage and continuity of hydrogen intake. This study evaluated the efficacy of a novel Si-containing agent that can generate substantial quantities of hydrogen to treat pressure ulcers in an in vivo mouse model. **Methods**: The back skin and subcutaneous tissue of mice were compressed with magnets for 12 h. Changes in the ulcer area after release of compression, histological findings, degree of apoptosis, and expression levels for oxidative stress markers and inflammation-related cytokines were compared between mice fed a normal diet (control group) and those fed a 2.5 wt% Si-based diet (Si group). **Results**: The Si group had a significantly smaller ulcer area and shorter healing period than the control group. Moreover, inflammatory responses, apoptotic activity, and oxidative stress within the ulcer tissue were suppressed significantly in the Si group. **Conclusions**: Oral intake of the Si-based agent can potentially treat and prevent pressure ulcers by regulating apoptosis, oxidative stress, and inflammatory responses.

## 1. Introduction

Pressure ulcers can be a major concern for patients and their caregivers and may burden the healthcare system as well [1]. Although individuals of all ages can be affected, pressure ulcers are more prevalent in the less active elderly population. In an aging society, the establishment of effective prevention and treatment methods has become increasingly critical.

The pathogenesis and exacerbation of pressure ulcers reportedly involve not only mechanical loads and ischemic damage but also ischemia/reperfusion injury (IRI) and inflammatory responses [1,2]. It is believed that IRI can arise from tissue oxidative stress associated with reactive oxygen species produced during blood flow interruption and subsequent reperfusion [3]. Suppression of IRI has been effective in managing pressure ulcers, and to this end, extensive research has focused on therapeutic agents exhibiting anti-inflammatory, antioxidant, and anti-apoptotic activities. For example, the therapeutic potential of vitamin E [4], melatonin [5], candesartan [6], olive oil [7], caffeic acid phenethyl ester [8], deferoxamine [9], derinat [10], sodium pyruvate [11], zinc [12], phospholipid fraction [13], dimethyl fumarate [14], baicalin [15] and tryptanthrin [16] for pressure ulcers has been reported in vitro or vivo studies. Furthermore, Zinc, arginine, and vitamin C, which are representative components possessing antioxidant properties, have demonstrated clinical efficacy [17]. These drugs can be used relatively safely, but concerns about side effects associated with overdose remain [18,19]. Particularly for elderly patients with underlying conditions—the primary group affected by pressure ulcers—there is a risk of more severe complications [20,21,22]. Therefore, careful dose adjustment and thorough monitoring are essential.

Hydrogen is a promising antioxidant that has been useful in reducing tissue oxidative stress by selectively neutralizing hydroxyl radicals [23], and its potential effectiveness against pressure ulcers has previously been reported [24,25]. Various hydrogen delivery systems, such as hydrogen water intake and hydrogen gas inhalation, have been explored to mitigate oxidative damage. However, most of those conventional studies used hydrogen water or hydrogen gas, which have limitations with regard to dosage and continuity of hydrogen intake. Hydrogen water is generally prepared by bubbling hydrogen gas into water or by using electrolysis. However, hydrogen solubility (saturation concentration: 1.6 ppm) in water is low and its concentration easily decreases over time when exposed to air because hydrogen molecules to diffuse rapidly into the atmosphere. Furthermore, maintaining continuous inhalation of hydrogen gas is impractical. Such limitations pose a significant challenge, particularly for chronic conditions like pressure injuries [26].

To overcome these limitations, a novel silicon (Si)-based agent that continuously releases molecular hydrogen in the intestinal environment after oral administration has been developed. This Si-based agent can produce over 400 mL/g of hydrogen within 24 h following oral administration, corresponding to an amount exceeding 22 L of fully saturated hydrogen water. The agent remains stable under ordinary conditions and can provide a sustained and high hydrogen yield in vivo. This delivery method offers advantages in dosage control, stability, and continuity of hydrogen exposure that are difficult to achieve with conventional hydrogen-water intake or hydrogen gas inhalation [26]. This agent has shown therapeutic efficacy in multiple models of oxidative stress-related disorders and IRI [27,28,29,30,31]. The present study aimed to evaluate its preventive and therapeutic effects against pressure ulcers in a mouse model.

## 2. Materials and Methods

### 2.1. Animal Model

The Animal Ethics Committee of the University of Osaka reviewed and approved the animal protocols (Approval No. 04-005-002, date: 8 April 2022). The Jackson Laboratory Japan, Inc. (Kanagawa, Japan) provided 27 male C57BL/6J mice (6 weeks old). Animals were kept under controlled laboratory conditions (12 h light/12 h dark; 22–25 °C) and given unrestricted access to water/food during experiments. In accordance with the 3Rs principle (Replacement, Reduction, and Refinement), the number of animals was minimized to achieve statistical validity, referencing past studies [13,29]. AIN-93 M (Oriental Yeast Co., Ltd., Tokyo, Japan) was used as the normal diet [32]. Based on previously established methods [26,33,34], a special diet was prepared by replacing 2.5% by weight of cornstarch in the normal diet with Si-based agent.

### 2.2. Study Design and Animal Pressure Injury Model

Mice were randomly categorized into 3 experimental groups: controls, pre-Si, and post-Si (*n* = 9 each). The control group received the AIN-93M diet throughout the study. The pre-Si group was administered the Si-based agent-containing special diet from 3 days before pressure administration to the study endpoint. In contrast, the post-Si group received the Si-based agent-containing diet following pressure release and continued on this diet until the study endpoint. The timing and duration of administration of the Si-based agent were based on previous studies [5,8,10,13,14,16].

**Figure 1:**
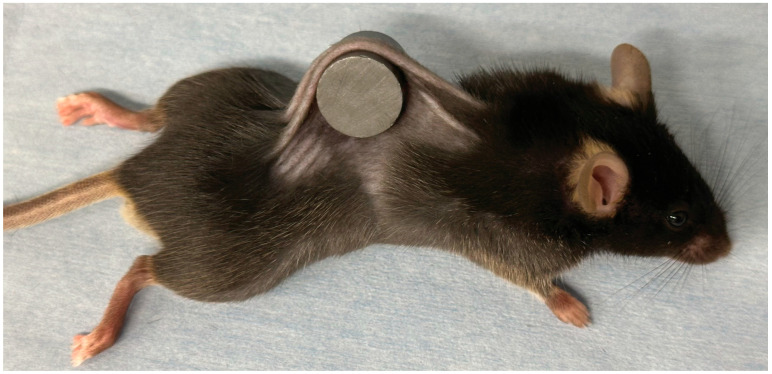
Mouse model of pressure ulcers. Pressure ulcers were established by applying 12-h compression to the dorsal skin using 2 magnetic plates.

The mouse pressure injury model was established using previously reported methods [12,13,14]. Mice were subjected to isoflurane inhalation (for anesthesia) and dorsal hair removal. The dorsal skin was elevated and compressed between 2 circular ferrite magnetic disks (12 mm diameter, 5 mm thickness, magnetic force: 1180 G; NeoMag Co., Tokyo, Japan), to ensure inclusion of the dermis, epidermis, loose connective tissue, and subcutaneous fat, while sparing the muscle (Figure 1). The magnetic disks were removed after 12 h of dorsal skin trapping. Compression was applied without further anesthesia or immobilization. Each mouse developed 2 distinct ulcers that were separated by unaffected skin. For the time-course analysis of the ulcer area, photographs of the ulcers were taken from 5 mice per group (10 ulcers) and assessed using ImageJ (version 2.9.0/1.53t) [35]. The remaining animals (*n* = 8 ulcers in each group) were euthanized by carbon dioxide inhalation on day 3 after pressure injury for tissue collection. The tissue from the center of the ulcer was fixed using 4% paraformaldehyde (FUJIFILM Wako Pure Chemical Corp., Osaka, Japan) for histological staining and the tissue from the adjacent area was stored at –80 °C for subsequent experimental procedures. All evaluations were conducted by investigators who were blinded to group allocation (outcome assessor blinding).

### 2.3. Histology

Samples were embedded in paraffin and then sectioned (4 µm thickness). Terminal deoxynucleotidyl transferase dUTP nick-end labeling (TUNEL) and hematoxylin & eosin (HE) staining were performed. For HE staining, one section per specimen was prepared, and histopathological evaluation was conducted in three different regions of each section. Histopathological evaluation based on HE staining followed established scoring criteria [6,36]. Specifically, tissue damage was assessed based on the integrity of the 3 skin layers and tissue loss depth around the ulcer centers (score of 0: there were no histological alterations in the epidermis, dermis, or subcutaneous tissue; score of 1: the 3 layers were intact but epidermal changes and fiber disruption were observed; score of 2: an epidermal defect was observed along with necrotic regions; score of 3: a dermal defect was observed along with necrotic regions; score of 4: a deep dermal defect was observed). Based on the manufacturer’s instructions, the Apoptosis Detection Kit (TaKaRa Bio Inc., Shiga, Japan) was used to assess apoptosis in situ via TUNEL staining. DAPI-containing Fluoro-KEEPER Antifade Non-Hardening Reagent (Nacalai Tesque Inc., Kyoto, Japan) was used to mount the sections. The following ratio (TUNEL-positive cells/DAPI-positive nuclei) was designated as the apoptotic index, as reported previously [14,29,31]. Microscopic imaging and quantitative analysis were conducted using the BZ-X800 microscope (Keyence, Osaka, Japan) with ImageJ software (version 2.9.0/1.53t).

### 2.4. Apoptosis-Related Protein Measurements

As previously described, expression levels for apoptosis-associated proteins were assessed using Western blots [13]. Frozen tissue samples were minced on ice, lysed using the EzRIPA Lysis kit (ATTO Co., Tokyo, Japan) with a homogenizer, and centrifuged. Supernatants were denatured with EzApply (ATTO Co., Tokyo, Japan), a total of 10 μg of protein per lane was loaded, subjected to electrophoretic separation using e-PAGEL HR (ATTO Co., Tokyo, Japan), and then transferred to PVDF membranes via the iBlot 2 Dry Blotting System (Invitrogen, Carlsbad, CA, USA). Membranes were immersed in blocking solution (TBST, 5% nonfat milk) for 30 min, and then incubated overnight with the primary antibodies at 4 °C. These included rabbit polyclonal anti-Bax (50599-2-Ig, 1:5000), anti-Bcl2 (26593-1-AP, 1:1000), and anti-β-actin (20536-1-AP, 1:1000). After membranes were washed, they were incubated at room temperature with goat anti-rabbit IgG (HRP-conjugated; SA00001-2, 1:2000) for 2 h (all antibodies acquired from Proteintech Group, Rosemont, IL, USA). Signal detection was conducted using EzWestLumi plus (ATTO Co., Tokyo, Japan), and the ChemiDoc Touch imaging system (Bio-Rad Laboratories, Hercules, CA, USA) was used to obtain images. The internal control was β-actin. The ratio of the apoptosis-promoting factor Bax to the inhibitory factor Bcl-2 was used for evaluating tissue apoptosis.

### 2.5. Oxidative Stress Measurements

For the evaluation of oxidative stress, DNA oxidation and lipid peroxidation were assessed by measuring levels for 8-hydroxy-2-deoxyguanosine (8-OHdG) and malondialdehyde (MDA), respectively, following previously reported methods [29,31]. The OxiSelect TBARS Assay Kit (Cell Biolabs, San Diego, CA, USA) was used to determine MDA levels, while the DC Protein Assay Kit (Bio-Rad Laboratories, Hercules, CA, USA) was used for total protein measurements. The DNA Extractor TIS Kit (FUJIFILM Wako Pure Chemical Corp., Osaka, Japan) was used for extraction of DNA, which was then hydrolyzed via the 8-OHdG Assay Preparation Set (FUJIFILM Wako Pure Chemical Corp., Osaka, Japan). The NanoDrop One spectrophotometer (Thermo Fisher Scientific, Waltham, MA, USA) was used for DNA concentration determinations. An extremely accurate ELISA assay (Japan Institute for Control of Aging, Nikken SEIL Co., Shizuoka, Japan) was used for quantification of 8-OHdG, and absorbance readings were obtained with the SH-9000Lab plate reader (HITACHI, Tokyo, Japan). Data were normalized to total protein or DNA content and presented as ng/mg DNA for 8-OHdG and nmol/mg protein for MDA.

### 2.6. Inflammatory Cytokine Gene Expression Measurements

mRNA expression levels for pro-inflammatory cytokines were assessed using real-time quantitative reverse-transcription PCR, otherwise known as RT-qPCR, based on previously reported methods [15,29,31]. Total RNA was prepared with the miTotal RNA Extraction Miniprep System (Viogene Biotek Corp., Taipei, Taiwan), and the ReverTra Ace qPCR RT Master Mix (TOYOBO, Osaka, Japan) was used for cDNA conversion. Expression levels for TNF-α (Mm00443258_m1), IL-6 (Mm00446190_m1), IL-1β (Mm00434228_m1), and β-actin (Mm02619580_g1) were evaluated using TaqMan Gene Expression Assays (Thermo Fisher Scientific, Waltham, MA, USA). qPCR was carried out with the THUNDERBIRD Probe qPCR Mix (TOYOBO, Osaka, Japan) on the QuantStudio 7 Flex Real-Time PCR System (Thermo Fisher Scientific, Waltham, MA, USA). Relative mRNA expression was normalized to β-actin with the 2–ΔΔCT method and quantified.

### 2.7. Statistics

Tukey’s Honestly Significant Difference test was used for statistical analysis along with JMP^®^ Pro 17 (SAS Institute, Cary, NC, USA). The normality of data distribution was assessed using the Shapiro–Wilk test. Statistical significance was reached when *p* < 0.05. Values were presented as mean ± standard error of the mean.

## 3. Results

### 3.1. Macroscopic Findings

**Figure 2:**
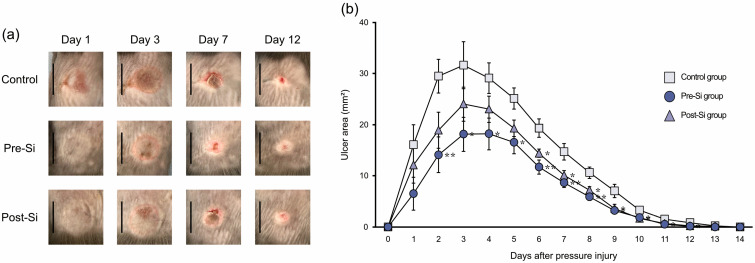
(**a**) Time course of macroscopic findings for pressure ulcers (scale bar = 10 mm). (**b**) Changes in ulcer area after pressure injury (** *p* <0.01, * *p* < 0.05; mean ± standard error indicated by bars; Tukey’s Honestly Significant Difference test; *n* = 10).

Ulcer formation was observed in all mice, and no mice were excluded from the analysis. At around day 3 after injury, the ulcers in all groups reached their maximal size, which then gradually decreased. By day 2, the pre-Si mice had significantly smaller ulcer areas (*p* < 0.05) than controls, and from day 6 onward, the post-Si group also exhibited significantly smaller ulcers than controls (Figure 2). In line with these findings, healing was accelerated in Si-treated mice. The mean healing times were 13.0 ± 0.2 days in controls, 11.8 ± 0.3 days in the pre-Si group, and 12.0 ± 0.3 days in the post-Si group, with both Si-treated groups healing significantly faster than controls (*p* < 0.05).

### 3.2. Histological Findings

**Figure 3:**
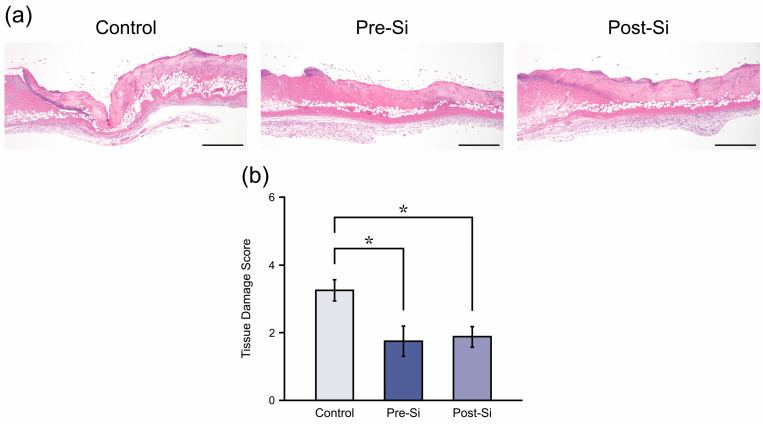
(**a**) Representative HE-stained sections at day 3 for each experimental group of mice (scale bar = 500 μm). (**b**) Tissue damage score for each group (* *p* < 0.05; mean ± standard error indicated by bars; Tukey’s Honestly Significant Difference test; *n* = 8).

At day 3, control ulcer tissues exhibited deep necrosis of the epidermis and dermis with dense inflammatory cell infiltration, whereas pre-Si and post-Si mice showed milder tissue damage (Figure 3a). Quantitative scoring of HE sections confirmed significantly lower tissue damage scores (*p* < 0.05) in both Si-treated groups in comparison to controls (Figure 3b), suggesting that histological injury was attenuated by the Si-based agent.

### 3.3. Apoptosis-Related Findings

**Figure 4:**
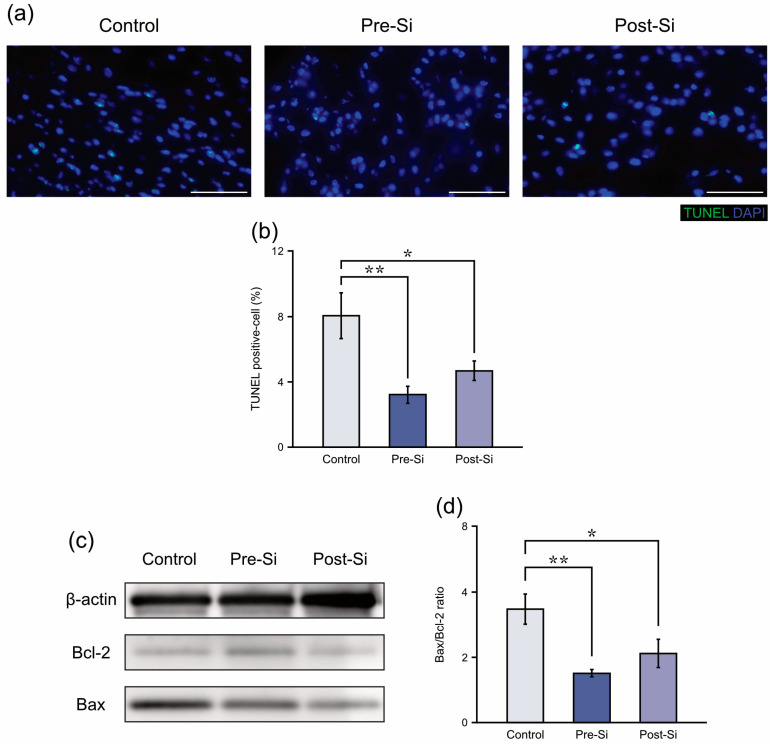
(**a**) Representative TUNEL-stained sections at day 3 for each experimental group of mice (scale bar = 50 μm). (**b**) Proportion (TUNEL-positive cells among DAPI-positive nuclei) for each experimental group of mice (** *p* < 0.01, * *p* < 0.05; mean ± standard error indicated by bars; Tukey’s Honestly Significant Difference test; *n* = 8). (**c**) Representative Western blot images for each experimental group. (**d**) Relative Bax/Bcl-2 ratio at day 3 for each experimental group of mice (** *p* < 0.01, * *p* < 0.05; mean ± standard error indicated by bars; Tukey’s Honestly Significant Difference test; *n* = 8).

TUNEL staining revealed that apoptotic cells accounted for 8.1 ± 1.4% of nuclei in control mice, compared with 3.2 ± 0.5% in pre-Si mice and 4.7 ± 0.6% in post-Si mice. Both Si-treated groups exhibited significantly fewer apoptotic cells than controls (post-Si: *p* < 0.05, pre-Si: *p* < 0.01) (Figure 4b). Similarly, there was a significant decrease in the Bax/Bcl-2 protein ratio in pre-Si and post-Si groups relative to controls (post-Si: *p* < 0.05, pre-Si: *p* < 0.01) (Figure 4d), according to the Western blot results. This suggests that apoptosis in pressure ulcer tissue was reduced by the Si-based agent.

### 3.4. Oxidative Stress Findings

**Figure 5:**
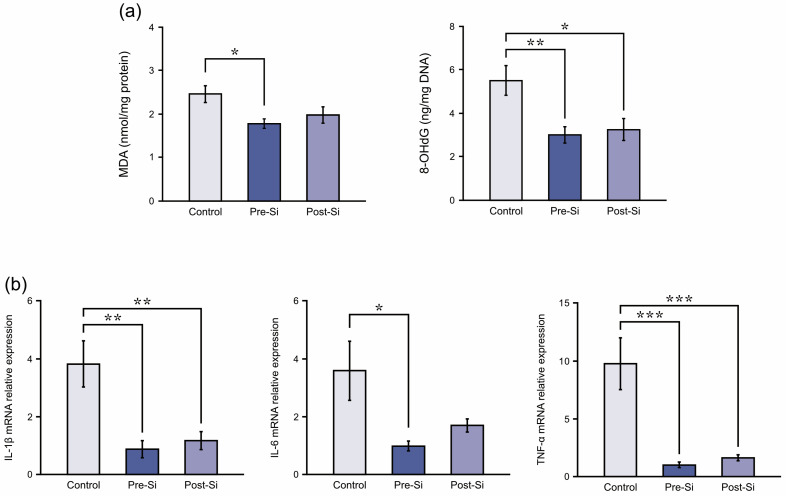
(**a**) Oxidative stress marker (malondialdehyde [MDA] and 8-hydroxy-2-deoxyguanosine [8-OHdG]) levels at day 3 for each experimental group of mice (** *p* < 0.01, * *p* < 0.05; mean ± standard error indicated by bars; Tukey’s Honestly Significant Difference test; *n* = 8). (**b**) Pro-inflammatory cytokine relative mRNA expression levels at day 3 for each experimental group of mice (*** *p* < 0.001, ** *p* < 0.01, * *p* < 0.05; mean ± standard error indicated by bars; Tukey’s Honestly Significant Difference test; *n* = 8).

Markers of oxidative stress were reduced in Si-treated mice (Figure 5a). Mean MDA levels (nmol/mg protein) were 2.5 ± 0.2 in controls, 1.8 ± 0.1 in pre-Si mice, and 2.0 ± 0.2 in post-Si mice, with the pre-Si group showing significantly lower levels than controls (*p* < 0.05). Mean 8-OHdG levels (ng/mg DNA) were 5.5 ± 0.7 in controls, 3.0 ± 0.4 in pre-Si mice, and 3.3 ± 0.5 in post-Si mice. Both Si-treated groups showed significantly reduced 8-OHdG levels compared with controls (*p* < 0.05).

### 3.5. Inflammation-Related Findings

Regarding pro-inflammatory cytokines, their relative mRNA expression was attenuated by Si treatment (Figure 5b). The pre-Si mice had significantly lower IL-1β, IL-6, and TNF-α levels (*p* < 0.01, *p* < 0.05, *p* < 0.001) compared with controls. The post-Si group also exhibited significantly decreased IL-1β and TNF-α levels (*p* < 0.01, *p* < 0.001), whereas the IL-6 reduction was not statistically significant. This suggests that inflammatory cytokine expression in ulcer tissue was reduced by the Si-based agent, and the most pronounced effects were observed under pre-treatment conditions.

## 4. Discussion

Our findings indicate that intake of the Si-based agent can effectively mitigate oxidative stress, inflammatory responses, and apoptotic activity in pressure ulcers, thereby contributing to a reduction in ulcer area and promoting early wound healing. Moreover, these effects were observed in both groups of mice that received oral administration starting before and after pressure injury, with no statistically significant differences between the pre-Si and post-Si groups, although the effects tended to be more pronounced in the pre-Si group. This supports the possible efficacy of the Si-based agent in both preventing and treating pressure ulcers.

Hydrogen reportedly has antioxidant, anti-inflammatory, and anti-apoptotic effects [37]. Furthermore, due to its rapid diffusion and non-toxic properties, hydrogen poses a minimal risk of adverse effects even when administered in excess [38]. This characteristic gives hydrogen an advantage over other therapeutic agents. In recent years, hydrogen has demonstrated protective effects against IRI across multiple organs [39] as well as pressure ulcers. Administration of hydrogen water through tube feeding decreased wound area and accelerated recovery in elderly individuals with severe pressure ulcers during hospitalization [24], while hydrogen inhalation reportedly inhibited pressure ulcer formation in mice [25]. On the other hand, methods for administering hydrogen to patients using hydrogen water or hydrogen gas have issues with regard to dosage and continuity of intake. Therefore, a more convenient and efficient method of hydrogen administration was sought.

The Si-based agent has shown therapeutic efficacy in a range of animal models for chronic diseases such as Parkinson’s disease, renal insufficiency [26], ulcerative colitis [33], and interstitial pneumonia [34], as well as acute oxidative stress-associated conditions, including IRI in the kidneys [28], skin [29], and intestines [30]. Moreover, hydrogen-based therapy using a Si-based agent has been proposed for conditions requiring prolonged treatment, which would be impractical with conventional hydrogen administration methods such as fat grafting [31]. Therefore, this agent may serve as an ideal preventive and therapeutic agent for conditions such as pressure ulcers, which involve a combination of early IRI after pressure injury and prolonged chronic inflammation. Furthermore, since pressure ulcers are more common in elderly people, the convenience of oral administration may be an advantage and because the Si-based agent itself is not absorbed by the body, concerns about overdose-related side effects are minimal. In fact, safety has been confirmed in past surveys, including a 91-day repeated-dose oral toxicity test (2000 mg/kg per day), the Ames test, chromosomal aberration test, and micronucleus test [26,31]. However, all previous reports on the Si-based agent have been based on animal studies. Therefore, clinical trials involving humans must be conducted in the future.

In addition to the pressure ulcers studied here, other refractory ulcers (e.g., radiation-induced dermatitis, diabetic ulcers, burn ulcers, venous stasis ulcers) often involve similar mechanisms of oxidative stress and chronic inflammation [40,41,42,43]. Regarding pressure ulcers and radiation-induced dermatitis, reports already suggest the potential efficacy of hydrogen-based treatments [44,45]. While no direct therapeutic effects of hydrogen have been reported for other chronic ulcers to date, hydrogen therapy, with its antioxidant and anti-inflammatory properties, may also be effective for these conditions. Treatment using Si-based agent has the potential to overcome the persistence limitations inherent in conventional hydrogen therapy. This approach could serve as an effective means to apply hydrogen therapy to chronic conditions requiring long-term treatment. Future research studies should focus on evaluating the therapeutic efficacy of the Si-based agent in managing these conditions.

This study has some limitations. First, variations in the preventive and therapeutic efficacy as a function of compression time or Si-based agent dose were not examined in this study. Second, the actual amount of hydrogen generated by intake of the Si-based agent and delivered to skin tissues could not be directly quantified. The compression time in the present mouse model was determined as 12 h based on prior studies [12,13,14]; however, the usefulness of the agent under prolonged or repeated pressure remains to be elucidated. In our investigation, the dietary concentration for the Si-based agent was selected based on previous reports [26,33,34]. The efficacy of this agent has been shown to be dose-dependent [33], which emphasizes the need for additional research to establish the optimal Si-based agent dosage across various disease models.

## 5. Conclusions

The Si-based agent effectively attenuated apoptotic processes, inflammatory responses, and oxidative stress within ulcerative tissue in a murine pressure ulcer model. The simple oral administration of this agent may prevent pressure ulcers and promote their early healing with few side effects, thus highlighting their value in future clinical practice.

## Data Availability

The data presented in this study are available from the corresponding author upon reasonable request.

## Abbreviations

The following abbreviations are used in this manuscript:

Si	Silicon
IRI	ischemia/reperfusion injury
HE	hematoxylin and eosin
TUNEL	terminal deoxynucleotidyl transferase dUTP nick end labeling
DAPI	4′,6-diamidino-2-phenylindole
PVDF	PolyVinylidene DiFluoride
TBST	Tris Buffered Saline with Tween 20
HRP	Horseradish Peroxidase
MDA	malondialdehyde
8-OHdG	8-hydroxy-2-deoxyguanosine
DNA	DeoxyriboNucleic Acid
RNA	RiboNucleic Acid
RT-qPCR	real-time quantitative reverse-transcription PCR
IL-1β	Interleukin 1 beta
IL-6	Interleukin 6
TNF-α	tumor necrosis factor alpha
ppm	parts per million
